# 

**Published:** 1916-01-01

**Authors:** 


### PASSING EVENTS AND TOPICS.

### A Unique Hospital.

ACCORDING to the *Sanitary Record* the Kitchener Indian Hospital at Brighton, equipped to accommodate over 2,000 patients, is unique in the entire absence of female assistance, even the laundry-work being done by Indian washermen specially brought over for the purpose.

### The Signing of Prescriptions.

AT the December meeting of the London Insurance Committee a recommendation was received from the Medical Benefit Sub-Committee to the effect that prescriptions issued by a practitioner acting as a deputy for a panel doctor must be signed in the name of the practitioner for whom the deputy is acting, followed by the initials of the deputy, and that prescriptions must not be signed in the name of a partnership.

### A Novel Scheme.

A SCHEME for providing a motor ambulance for the Front from the proceeds of the sale of medicine bottles collected from houses in Islington is said to be making good progress. Boy Scouts and other voluntary workers are engaged in the house-to-house collection of empty bottles, but more helpers are needed. Mr. H. Blok, 7 Carleton Road, Tufnell Park, is the originator of the scheme, and would be glad to get more helpers.

### Pennies and their Possibilities.

SOME few months ago fourteen boys and girls living in the district of Highams Park were given one penny each and asked to use it in any way they could devise in order to raise funds for the local Red Cross Detachment, with the result that when it was asked that the various moneys should be returned, no less than the sum of £2 15s. 7d. was found to have been realised. Many and varied methods have been employed by the children to earn money, one girl, profiting by the "kill that fly" crusade and its subsequent precautionary measures, devoted all her energies to the making and selling of muslin milk-covers, and easily headed the list of amounts obtained with the sum of fifteen shillings.

### The "London Hospital Gazette."

THE December number of the *London Hospital Gazette* is unusually interesting and well illustrated. Articles contributed from various parts of the world show how old "London" men are scattered in practically all the spheres of the present war. The lists of past students of this hospital who are now serving with the Forces occupies fourteen pages, and a long list is also given of the members of the nursing staff who are with the Navy and Army Nursing Reserve. The names are given of twenty-three "London" men who have fallen in the war.

### The Surtax on Immature Spirit.

AT last the Commissioners of Customs and Excise have made regulations laying down the conditions under which hospital authorities and other users of medicinal spirits may claim repayment of the surtax (1s. 6d. per proof gallon) on immature spirit used in the manufacture of tinctures and other compounds. Applications for repayment must be made through the local officials of Customs and Excise, and applicants must be in a position to produce the permits received with the spirit, together with accounts showing the date of receipt of the spirit and the date of use, with the actual purpose to which the spirit has been applied. No repayment will be made in respect of spirit used in the preparation of compounds employed mainly as flavouring agents.

### Personalia.

MR. NATHANIEL BISHOP HARMAN, M.A. (Cantab), M.B., B.C., F.R.C.S., has been appointed ophthalmic surgeon to the West End Hospital for Diseases of the Nervous System.

MR. MAURICE GEPP, L.R.C.P., L.R.C.S. Edin., has been appointed medical officer of health to the combined districts comprising the boroughs of Bishop's Castle and Wenlock, Church Stretton Urban District, and Atcham, Church Stretton, and Clun Rural District.

DR. FRANCIS T. HEUSTON, F.R.C.S.I., of St. Stephen's Green, Dublin, whose death has recently been announced, was one of the leading surgeons in Ireland. He was formerly Professor of Anatomy in the Royal College of Surgeons, Ireland, and consulting surgeon to various Dublin hospitals.

OWING to absence on military duty Mr. O. E. Warburg has found it necessary to resign his chairmanship of the Public Health Committee of the London County Council. Mr. F. R. Anderton has been appointed chairman in his stead, and Mr. R. C. Norman has been appointed a member of the committee.

THE following members of the London County Council's staff have been given authority to enter and inspect any premises used or believed to be used for the purposes of a lying-in home: Dr. Elizabeth Macrory, Dr. Mary A. Pilliet, Mr. H. A. Jury, Mr. E. C. Browne, Mr. J. Court, Miss I. G. Smith, Miss F. E. H. Marshall, Miss A. M. Bell, Miss M. D. Drieselman, Miss M. J. Phillips, Miss E. O'Donnell, Miss M. M. Keohane, Miss M. E. Lockington, Miss E. A. Addison, and Miss E. S. Stone.

### The Book Market.

LIST OF NEW BOOKS RECEIVED FROM THE FOLLOWING PUBLISHERS:—

A. and C. Black: "The Structure of the Fowl." By A. Charnock Bradley, M.D., M.R.C.V.S. 3s. 6d. net.

Cassell and Co.: "The Seven Ages of Woman." By Mary Scharlieb, M.D., M.S. 6s. net.

The Oxford University Press: "The Empire Birthday Book." 1s. net.

Hodder and Stoughton: "The Queen's Gift Book." 2s. 6d. net.

### Institutional and Public Reports.

Shaftesbury Society and Ragged School Union: "Out of the House of Bondage." The seventy-first Annual Report of the Union.

Dr. R. W. Johnstone's Report to the Local Government Board on the Progress and Diffusion of Plague, Cholera, and Yellow Fever throughout the World during 1913. Price 1s.

Lord Mayor Treloar Cripples' Hospital and College, Alton: First Medical Report, by H. J. Garvain, B.C., Medical Superintendent.

### Scientific Papers.

National Health Insurance. Medical Research Committee. Full Report of the Special Investigation Committee upon the Incidence of Phthisis in Relation to Occupations. The Boot and Shoe Industry. (Wyman and Sons, Ltd. 1915. Price 3d.)

RATES OF SUBSCRIPTION: Twelve months, 6/6; Six months, 4/-; Three months, 2/-, post free to any address in the United Kingdom. Abroad, Twelve months, 8/8; Six months, 5/-; Three months, 2/6, post free.—Address The Subscription Dept., "THE HOSPITAL," The Hospital Building, 28/29 Southampton Street, Strand, London, W.C.

